# Single-cell transcriptomic analysis reveals dynamic activation of cellular signaling pathways regulating beige adipogenesis

**DOI:** 10.1038/s12276-024-01252-9

**Published:** 2024-10-28

**Authors:** Dong Soo Kyung, Eunmin Lee, Sehyun Chae, Yeonho Son, Ye-Jin Moon, Daehee Hwang, Jong Kyoung Kim, Yun-Hee Lee, Je Kyung Seong

**Affiliations:** 1 Laboratory of Developmental Biology and Genomics, College of Veterinary Medicine, Interdisciplinary Program for Bioinformatics and Program for Cancer Biology, Seoul National University, Seoul, 08826 Republic of Korea; 2 Department of New Biology, DGIST, Daegu, 42988 Republic of Korea; 3 Division of Chemical Engineering and Bioengineering, College of Art, Culture and Engineering, Kangwon National University, Chuncheon, 24341 Republic of Korea; 4 College of Pharmacy and Research Institute of Pharmaceutical Sciences Seoul National University, Seoul, 08826 Republic of Korea; 5 School of Biological Sciences, Seoul National University, Seoul, 08826 Republic of Korea; 6 Department of Life Sciences, Pohang University of Science and Technology (POSTECH), Pohang, 37673 Republic of Korea

**Keywords:** Computational biology and bioinformatics, Obesity

## Abstract

PDGFRA+ cells have been identified as adipocyte stem cells (ASCs) that differentiate into beige adipocytes in white adipose tissue (WAT) following thermogenic stimuli. To elucidate the molecular heterogeneity of ASCs, we conducted single-cell transcriptomic profiling of PDGFRA+ cells isolated from the inguinal WAT (iWAT) of mice treated with the beta3 adrenergic receptor agonist CL316243. Single-cell RNA-seq revealed nine major clusters, which were categorized into four groups: resting, proliferating, differentiating, and adipogenic factor-expressing cells (AFECs). Trajectory analysis revealed sequential activation of molecular pathways, including the Hedgehog and Notch signaling pathways, during beige adipogenesis. AFECs expressed *Dpp4* and did not differentiate into adipocytes in culture or after transplantation. Furthermore, genetic lineage tracing studies indicated that DPP4+ cells did not differentiate into adipocytes in iWAT during CL316243-induced beige adipogenesis. However, high-fat diet feeding led to the recruitment of adipocytes from DPP4+ cells in iWAT. Overall, this study improved our understanding of the dynamic molecular basis of beige adipogenesis and the potential contribution of DPP4+ adipocyte lineages to the pathological expansion of WAT during diet-induced obesity.

## DPP4+ Adipocyte Lineages: Role in Diet-Induced Adipose Tissue Expansion

This research examines beige adipogenesis, or the creation of ‘beige’ fat cells that burn energy and could help fight obesity. The scientists discovered a group of cells, identifed by specific markers PDGFRA and DPP4, which serve as a source for beige adipogenesis but don’t turn into beige fat cells themselves. They also found that these cells can change to become fat cells under certain situations, like a high-fat diet. The study also showed that the Hedgehog and Notch signaling pathways are vital in the transformation of PDGFRA+ cells into beige fat cells. These discoveries could be important for developing anti-obesity therapeutics.

This summary was initially drafted using artificial intelligence, then revised and fact-checked by the author.

## Introduction

Adipose tissue is a major metabolic organ that can be subcategorized into two types: white (WAT) and brown adipose tissue (BAT)^1^. WAT specializes in storing and mobilizing lipids, while BAT is responsible for nonshivering thermogenesis^1^. Although brown and white adipocytes are known to originate from distinct mesenchymal stem cell lineages, white adipocytes can be converted into brown-like adipocytes, called beige adipocytes (BAs), under thermogenic stimuli, such as cold temperature and beta-adrenergic stimuli^1^. This process, known as WAT browning, is characterized by the induction of uncoupling protein 1 (UCP1) and an increase in oxidative mitochondrial metabolism; the latter is beneficial for increasing energy expenditure and improving metabolic dysfunction in obesity-related disease models^1^.

In addition to direct conversion from white adipocytes, BAs are thought to be derived from the de novo differentiation of adipocyte progenitors. Several markers of adipocyte progenitors in inguinal WAT (iWAT) include early B-cell factor 2 (EBF2)^2^, platelet-derived growth factor receptor alpha (PDGFRA)^3^, transient receptor potential cation channel subfamily V member 1 (TRPV1)^4^, and platelet-derived growth factor receptor beta (PDGFRB)^5^. Among these markers, PDGFRA is expressed in multiple subpopulations with different levels of adipogenic potential and commitment statuses. For example, CD24+ PDGFRA+ cells become CD24− PDGFRA+ progenitors when they are committed to the adipocyte lineage^6^. While EBF2+ PDGFRA+ and EBF2− PDGFRA+ cells undergo de novo adipogenesis, only EBF2+ PDGFRA+ cells activate a thermogenic program in response to rosiglitazone^2^. These data suggest the cellular heterogeneity of PDGFRA+ adipocyte progenitors as they are not synchronized with respect to adipogenic potential.

Previously developed approaches using genetic tracing and flow cytometry require a selection of limited markers to tag and trace cells. Although these approaches can be used to effectively identify cell types that contribute to tissue plasticity, these methods provide noncomprehensive results and biased analyses of the employed markers. Accordingly, studies assessing the heterogeneity of PDGFRA+ progenitors and inferring their differentiation trajectories have been limited. Recently, single-cell mRNA sequencing (scRNA-seq) has been employed to acquire information that is largely unbiased and far more comprehensive than that obtained via previous approaches^7^. Several studies using scRNA-seq have characterized the heterogeneous molecular phenotypes of adipocyte progenitors during development^8^ and under beta-adrenergic stimulation^7^, cold exposure^9^, and high-fat diet feeding^10^. However, in-depth characterization of the cellular heterogeneity of PDGFRA+ progenitors during beige adipogenesis in iWAT has not yet been performed.

Here, we performed scRNA-seq of PDGFRA+ progenitors isolated from subcutaneous iWAT with or without beta3 adrenergic receptor activation. Various analyses identified subpopulations of PDGFRA+ progenitors, developmental trajectories during de novo differentiation, and dynamic signaling cascades underlying developmental transitions along the trajectories.

## Materials and methods

### Animals

All animal protocols were approved by the Institutional Animal Care and Use Committees at Seoul National University (SNU-200302-3, SNU-191113-2, and SNU-191022-2). Mice were fed a standard chow diet, housed at 22 °C, and maintained on a 12-h light/12-h dark cycle with free access to food and water at all times. Male mice were used for the experiments. C57BL/6 mice (6 weeks old, male) were purchased from the Central Lab. Animal, Inc (Seoul, Korea). PDGFRA-Cre [stock #013148, C57BL/6-Tg(PDGFRA-Cre)1Clc/J], PDGFRA-CreER [stock #018280, B6N.Cg-Tg(PDGFRA-Cre/ERT) 467Dbe/J], ROSA26-LSL-ZsGreen [stock #007906, B6.Cg-Gt(ROSA)26Sortm6(CAG-ZsGreen1)Hze/J], and ROSA26-LSL-tdTomato [stock#007909, B6.Cg-Gt(ROSA)26Sortm9(CAG-tdTomato)Hze/J] mice were purchased from the Jackson Laboratory (Bar Harbor, ME, USA). DPP4-CreER [stock#RMRC13230] mice were purchased from NARLabs (National Laboratory Animal Center, Taipei, Taiwan). DPP4-CreER, PDGFRA-Cre, and PDGFRA-CreER mice were crossed with ROSA26-LSL-tdTomato or ZsGreen mice to trace DPP4+ cells and PDGFRA+ cells during CL treatment or for transplantation assays. For Cre recombination, double transgenic mice and wild-type controls were treated with tamoxifen dissolved in sunflower oil (75 mg/kg; Sigma‒Aldrich, St. Louis, MO, USA) by oral gavage every 5 consecutive days^3^. Additionally, we included vehicle (oil)-treated control groups to confirm the specific induction of Cre recombinase activity in adipose tissues upon tamoxifen treatment. Experiments were started 10 days after the last dose of tamoxifen. For beta3 adrenergic receptor stimulation, mice were treated with CL316243 (1 mg/kg/day; Sigma‒Aldrich) by intraperitoneal injection for up to 7 days. For EdU labeling of proliferating cells, mice were injected with EdU (0.4 μmol/mouse; Thermo Fisher, Waltham, MA, USA) at the indicated times. For the transplantation assay, PDGFRA+ DPP4+ and PDGFRA+ DPP4− cells were isolated from PDGFRA-tdTomato mice and subcutaneously injected with Matrigel (Corning Inc., Corning, NY, USA), as described previously^3^. Matrigel transplants were collected and analyzed 3 weeks after injection.

### Cell cultures

C3H10T1/2 cells (ATCC, Manassas, VA, USA) were cultured as previously described^11^. For genetic modulation using siRNA, cells were transfected with 20 nM predesigned siRNA targeting *Gli1* (Bioneer, Daejeon, Korea, 14632) or *Rbpj* (Bioneer, 19664) or negative control siRNA (Bioneer, SN-1013) using INTERFERin® transfection reagent (Polyplus, Illkirch-Graffenstaden, France, #409-10) according to the manufacturer’s instructions. For overexpression of GLI1 and the GFP-tagged GLI2 protein, pLUT7-HA-GLI1 (Addgene, Watertown, MA, USA, plasmid #62967) and the pCEFLmGFP-Gli2 vector (Addgene, plasmid #37672) were obtained and transfected into the cells by using a JetPrime Transfection Kit (Polyplus, #114-15) according to the manufacturer’s instructions. For inhibition of DPP4 activity, sitagliptin (Sigma‒Aldrich) was used.

### WAT stromovascular cell fractionation and FACS analysis

For flow cytometry analysis, stromovascular cell (SVC) fractions from mouse iWAT were isolated as described previously^3^ and used for scRNA-seq analysis. For EdU detection, fixed SVCs were first processed for the Click-it reaction (ClickTech EdU Cell Proliferation Kit; Thermo Fisher) according to the manufacturer’s instructions, followed by cell surface marker staining. For cell surface marker staining, the following antibodies were used: anti-PDGFRA-APC (rat, 1:50; Biolegend, San Diego, CA, USA), anti-DPP4-FITC (rat, 1:50; Biolegend), anti-CD45-Pacific Blue (rat, 1:200; Biolegend), anti-NOTCH1-APC (hamster, 1:100; Biolegend), anti-PTCH1 (goat, 1:100; R&D Systems, Minneapolis, MN, USA), and donkey anti-goat IgG-Alexa Fluor 488 (1:200; Invitrogen, Waltham, MA, USA). Analytic cytometry was performed using a BD FACS LSRFortessa X-20 or FACS Lyric system (BD Biosciences, Franklin Lakes, NJ, USA). A FACSAria III system (BD Biosciences) was used to sort the cell surface marker-labeled cells. The raw data were processed using FlowJo software v.10 (TreeStar Inc., Ashland, OR, USA). The sorted cells were plated and cultured at an initial concentration of 1$\times$10^5^ cells/ml in growth medium and then switched to adipogenic induction medium, as described previously^3^.

### Histology

Adipose tissue was processed to obtain histological sections, and 5 μm-thick paraffin sections were subjected to immunohistochemical analysis as previously described^3^. EdU analysis was performed according to the manufacturer’s instructions (Click-iT™ Plus EdU Alexa Fluor™ 488 Imaging Kit; Thermo Fisher). Species-matched IgGs were used or the primary antibodies were omitted as nonspecific controls for immunohistochemistry. All quantitative analyses of histologic samples was carried out in a blinded manner. The antibodies used for immunohistochemistry included tdTomato (rabbit, 1:100; mouse, 1:100; Clontech, Tokyo, Japan), UCP1 (0.5 mg/ml rabbit; Abcam, Cambridge, United Kingdom), PDGFRA (goat, 1:200; R&D Systems), PTCH1 (goat, 1:100; R&D Systems), and NOTCH1 (hamster, 1:50; Biolegend). For lipid staining, BODIPY or LipidTOX (Invitrogen) was used. For nuclear staining, DAPI (Sigma‒Aldrich) was used.

### Analysis of mRNA expression levels

RNA was extracted using TRIzol® reagent (Invitrogen), and 1 μg of RNA was reverse transcribed using a cDNA synthesis kit (High-capacity cDNA Reverse Transcription Kit; Applied Biosystems, Waltham, MA, USA). One hundred nanograms of cDNA was subjected to qRT‒PCR in 20-μl reaction volumes (iQ SYBR Green Supermix; Bio-Rad, Hercules, CA, USA) with 100 nM primers. qRT‒PCR was performed using SYBR Green dye and a CFX Connect Real-time system (Bio-Rad) for 45 cycles, and the fold change in expression for all the samples was calculated by using the 2^−ΔΔCt^ method. Peptidylprolyl isomerase A (PPIA) was used as a housekeeping gene for mRNA expression analysis. The primers used for qRT‒PCR are listed in Supplementary Table 2.

### Oil red O staining

For lipid droplet staining, Oil Red O staining was performed according to the manufacturer’s protocol. Briefly, cells were fixed with 4% paraformaldehyde for 30 min, washed with 60% isopropanol, and stained with Oil Red O (0.5% Oil Red O; Sigma‒Aldrich) in 60% isopropanol. The stain was extracted in isopropanol and measured at 492 nm using a microplate spectrophotometer (MultiSkan GO, Thermo Fisher).

### Dpp4 promoter reporter analysis

HEK293T cells (ATCC) were transfected with DPP4 reporter vectors (MPRM34034; Genecopoeia, Rockville, MD, USA) with a JetPrime transfection kit. Additionally, pBABEPuro-HA-GLI1 (Addgene plasmid #62967), pLPCX-NICD1 (Addgene plasmid #44471), or pUC19 control plasmids (TaKaRa Bio, Tokyo, Japan) were cotransfected as indicated. At 24 h post-transfection, the cells were treated with either the Hedgehog inhibitor GANT61 (Cayman, Ann Arbor, MI, USA) or the gamma-secretase inhibitor DAPT (Cayman) to suppress Hedgehog and Notch signaling, respectively. After 24 h, the cell culture media were collected, and Gaussia luciferase (GLuc) and secreted alkaline phosphatase (SeAP) activities were measured via a Secrete-Pair Dual Luminescence Assay Kit (Genecopoeia) according to the manufacturer’s instructions. Ten microliters of media was used for each luminescence reaction. Luminescence signals were measured using a microplate luminometer (Centro LB 960, Berthold Technologies USA LLC, Oak Ridge, TN, USA). GLuc luciferase activity was normalized to SeAP activity.

### Hedgehog and notch signaling reporter analysis

For stable cell line establishment, HEK293T cells were transfected with pMuLE_EXPR_CMV-eGFP_TOP-NLuc1.1_12GLI-FLuc_CBF-GLuc (Addgene, plasmid #113862), pMD2.G (Addgene, plasmid #12259), and psPAX2 (Addgene, plasmid #12260) at a ratio of 4:1:3 using a JetPrime transfection kit. Lentiviral particles were collected over 2 days, and C3H10T1/2 cells were transduced with the particles and polybrene (Sigma‒Aldrich). Following transduction, the cells were selected with G-418 (AG Scientific, San Diego, CA, USA) for one week. Alternatively, transient transfection of pMuLE_EXPR_CMV-eGFP_TOP-NLuc1.1_12GLI-FLuc_CBF-GLuc into HEK293T cells was performed. For the GLuc assay, 10 μl of medium (without lysis) was added to 96-well white-bottom microplates, and 100 μl of coelenterazine buffer (Genecopoeia) was added. For the firefly luciferase (FLuc) assay, cells were lysed in Reporter Lysis Buffer (Promega) and centrifuged at 10,000 × *g* for 10 min at 4 °C, after which the supernatant was collected for analysis. Ten microliters of sample was loaded on 96-well white-bottom microplates, and 50 μl of luciferin buffer (Promega) was subsequently added. The luciferase activity of each construct was analyzed using a microplate luminometer (Centro LB 960). Luciferase activity values were normalized to the protein concentration of the lysate.

### scRNA-seq experiments

SVC fractions were isolated from mouse iWAT, and PDGFRA+ cells were subsequently enriched from the fractions using FACS, as described previously^3^. The sorted cells were stained with Trypan blue and resuspended at a concentration of 1 × 10^5^ to 2 × 10^6^ cells/ml. Cell viability was estimated to be greater than 80%. Using these cells, we performed single-cell library preparation according to the manufacturer’s protocol using the Chromium Single-cell 3′ Reagent Kit v2 (10× Genomics, Pleasanton, CA, USA). Briefly, cell suspensions were loaded onto each channel of a Chromium Single Cell A Chip along with reverse transcription master mix and single-cell 3′ gel beads to capture 3000 cells per channel. Gel beads-in-emulsions (GEMs) were generated by running the Chromium Controller, followed by reverse transcription using a Mastercycler Nexus system (Eppendorf AG, Hamburg, Germany). cDNA was amplified (12 PCR cycles) and purified using SPRIselect beads (Beckman Coulter, Brea, CA, USA). Purified cDNA was processed sequentially for enzymatic fragmentation, end repair, and A-tailing. Sequencing libraries with unique sample indices were pooled for multiplexing and subsequently sequenced using the Illumina HiSeq 2500 platform in high-output mode.

### scRNA-seq data processing

The Cell Ranger software suite (v2.0.0) provided by 10X Genomics was used to demultiplex sample barcodes, process cell barcodes (CBs) and UMIs, and construct a gene-by-cell count matrix. The raw BCL files were demultiplexed into FASTQ files using the cellranger mkfastq in Cell Ranger software. For each sample, paired-end reads in the FASTQ files were reformatted for processing CBs and UMIs, mapped to the mouse reference genome (GRCm38) by STAR (v2.5.2b) with an Ensembl GTF file (release 89), and quantified using CellRanger count in Cell Ranger software with the option of the expected number of recovered cells set to 3000 (--expect-cells = 3000). The output files of each sample were aggregated into a single gene-by-cell count matrix without normalization using cellranger aggr. We then filtered out CBs that corresponded to empty droplets after determining a threshold (called “a knee point”) in the barcode rank plot. All CBs with a total UMI less than the knee point were discarded. Next, we removed low-quality cells that had greater than 10% UMIs associated with mitochondrial-encoded genes and greater than 99.5% of the genes not expressed. The thresholds were chosen by visually inspecting outliers in the 2-dimensional principal component analysis (PCA) score plots of cell-level quality control (QC) metrics calculated by the scater R/Bioconductor package (v1.4.0), as suggested by Ilicic et al.^12^. Finally, for removal of cell-specific biases in the combined gene-by-cell count matrix, the raw counts were normalized by pooled size factors that were estimated from the scran R/Bioconductor package (v1.5.8). The normalized counts were then log2-transformed with a pseudocount of 1.

### scRNA-seq data analysis

Highly variable genes (HVGs) were identified using the trendVar and decomposeVar functions of the scran package with a false discovery rate (FDR) ≤ 0.05 and biological variability >0.1. Because the combined dataset consists of two technical batches, the batch information was incorporated into a design matrix, which was subsequently used as an input into the trendVar function. To visualize each cell in a 2-dimensional space, we reduced the dimensionality using the t-SNE algorithm implemented in the RunTSNE function of the Seurat package (v2.1.0) with the first 20 principal components (PCs) obtained for the HVGs and perplexity = 30. To filter out immune cells based on hematopoietic markers, we grouped cells into 4 clusters using the k-means clustering algorithm. The cells were further grouped into 24 clusters using the FindClusters function of the Seurat package with the first 50 PCs for the HVGs. Of the 24 clusters, immune-related clusters identified via previous k-means clustering were filtered out for downstream analysis.

After analysis of HVGs in nonimmune cells as described above, cells were visualized in a 2-dimensional t-SNE plot using the RunTSNE function of the Seurat package with the first 50 PCs and perplexity = 30. For clustering, we used the FindClusters function of the Seurat package with the first 50 PCs on the HVGs. The initial marker genes of each cluster were identified using the Wilcoxon rank sum test in the FindAllMarkers function of the Seurat package with an adjusted *P* value < 0.05 and a fold change >1.3. We then applied partial least square-discriminant analysis (PLS-DA) to the initial marker genes and computed the variable importance in projection (VIP), which represents the relative contribution of each gene to the separation of a cluster from the other clusters^13^. For each cluster, to determine a cutoff of significant VIPs, we estimated an empirical distribution of the null hypothesis for VIPs (i.e., a gene has no contribution to the separation) by applying the Gaussian kernel density estimation method^14^ to VIPs resulting from 1000 random permutations of cells. The adjusted *P* values of the VIPs of the individual genes were computed by the one-tailed test using the empirical null distribution. Finally, among the marker genes selected for each cluster by the rank sum test, we further selected the final marker genes as genes with an adjusted *P* value < 0.05 according to the VIP test.

For trajectory inference analysis, we performed feature selection by identifying HVGs in cells belonging to the Progen, Prolif, and Diff groups and clustering these cells with the HVGs as described above. We selected the union of the marker genes across all clusters as a set of feature genes. We then normalized the raw counts by the scran size factor and reduced the dimension of the normalized matrix of the feature genes to 2 using the DDRTree method in the Monocle (v2.6.1)^15^ package. For each trajectory, we ordered cells along the trajectory using the orderCells function of the Monocle package. Finally, the union of the marker genes was clustered based on their smoothed expression profiles along the trajectory using the plot_pseudotime_heatmap function of the Monocle package, and the genes showing early, middle, or late upregulation along each trajectory were identified based on the clustering results. For each gene, the smoothed relative gene expression profile along each of the trajectories was plotted using the RollApply function of the zoo R package (v1.8-3) with a window size of 150 (control) or 300 (CL-treated).

### Enrichment analysis of GO biological processes and pathways

Functional enrichment analysis of the marker genes was performed using DAVID software^16^. The enrichment of Gene Ontology biological process (GOBP) and Kyoto Encyclopedia of Genes and Genomes (KEGG) pathways associated with the marker genes was considered to be associated with *P* < 0.05.

### Network analysis

The network model for the selected marker genes was visualized using Cytoscape^17^. The nodes in the network model were arranged based on their locations in their associated pathways in the KEGG pathway database, and the edges in the network model were defined to display their activation/inhibition information obtained from the KEGG pathways.

## Results

### Beta 3 adrenergic receptor stimulation leads to de novo beige adipogenesis of PDGFRA+ progenitors

**Fig. 1:**
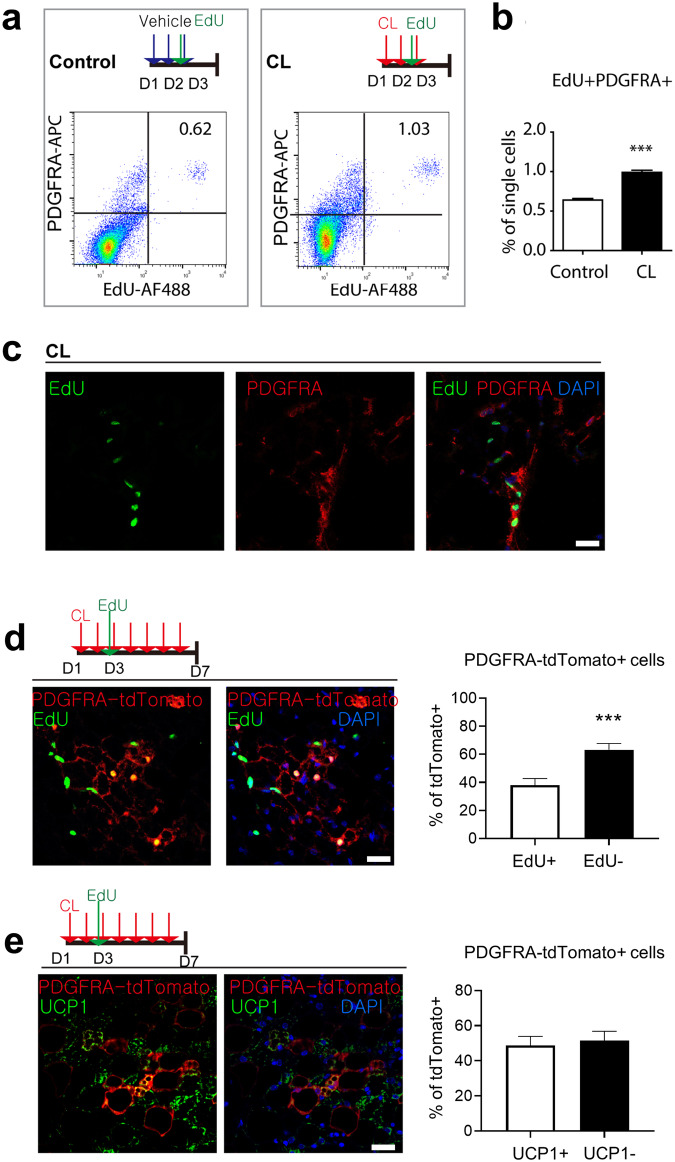
Proliferation and de novo differentiation of PDGFRA+ progenitors after CL stimulation. **a** Flow cytometric analysis of PDGFRA expression and EdU incorporation. **b** Quantification of the percentages of EdU+ PDGFRA+ cells under control and CL treatment conditions. **c** Images of EdU and PDGFRA staining in paraffin sections of iWAT from mice treated with CL for 3 days and injected with EdU 4 h before analysis. Bar = 25 μm. **d** Immunostaining of EdU and tdTomato in paraffin sections of iWAT from PDGFRA-CreER/tdTomato reporter mice treated with CL for 7 days and injected with EdU on Day 3. Bar = 25 μm. **e** Immunostaining of UCP1 and tdTomato in paraffin sections of iWAT from PDGFRA-CreER/tdTomato reporter mice treated with CL for 7 days and injected with EdU on Day 3. Bar = 25 μm. The data were analyzed by an unpaired two-tailed *t* test (mean ± SEM, *n* = 5–8; ***P < 0.001).

Previously, PDGFRA+ cells in WAT were identified as adipocyte progenitors that proliferate and differentiate into beige adipocytes after treatment with the beta3 adrenergic receptor agonist CL316243 (CL)^3^. Although gonadal WAT (gWAT) has a greater mitotic response of PDGFRA+ cells than does iWAT, iWAT can recruit more UCP1+ beige adipocytes after CL treatment; thus, we investigated the molecular characteristics of de novo beige adipogenesis of PDGFRA+ progenitors in iWAT after CL treatment. For analysis of the proliferation of PDGFRA+ cells in iWAT, C57BL/6 mice were injected with CL for 3 days and labeled with EdU 4 h before analysis of the mitotic index. Flow cytometry analysis confirmed that CL treatment significantly (*P* < 0.001) increased the number of EdU+ PDGFRA+ cells in iWAT compared to that in control conditions (Fig. 1a, b). Consistently, immunohistochemistry showed EdU incorporation in PDGFRA+ cells in the iWAT of the mice treated with CL for 3 days (Fig. 1c). Next, we traced the fate of PDGFRA+ cells during one week of CL treatment via lineage tracing studies using PDGFRA-CreER/tdTomato reporter lines, as previously reported^3^. Nearly one-third of the PDGFRA-tdTomato+ multilocular adipocytes (37%) in iWAT were positive for EdU (Fig. 1d), indicating that these newly generated EdU+ adipocytes originated from PDGFRA+ cells and proliferated on Day 3 of CL treatment. Moreover, 48% of the PDGFRA-tdTomato+ adipocytes were UCP1+ (Fig. 1e), indicating that half of the adipocytes newly differentiated from progenitors became UCP1+ beige adipocytes in the iWAT. Collectively, these data indicated that PDGFRA+ cells in iWAT undergo proliferation and de novo adipogenesis to BAs.

### scRNA-seq reveals the cellular heterogeneity of CL-treated PDGFRA+ progenitors

To investigate the cellular heterogeneity of PDGFRA+ progenitors, we performed scRNA-seq analysis of PDGFRA+ cells that were isolated from the iWAT of the mice injected with control vehicle or CL for 3 days. Isolation of PDGFRA+ cells and generation of sequencing libraries were performed separately for independent experiments conducted in triplicate of the controls (*n* = 3) and CL-treated mice (*n* = 3) (Supplementary Fig. 1a). After sequencing the pooled library, we selected 2676 and 8686 PDGFRA+ cells with an average of 2543 genes and 3816 unique molecular identifiers (UMIs) per cell for the control and CL-treated conditions, respectively, based on previously reported quality control criteria (Supplementary Fig. 1b–m).

**Fig. 2:**
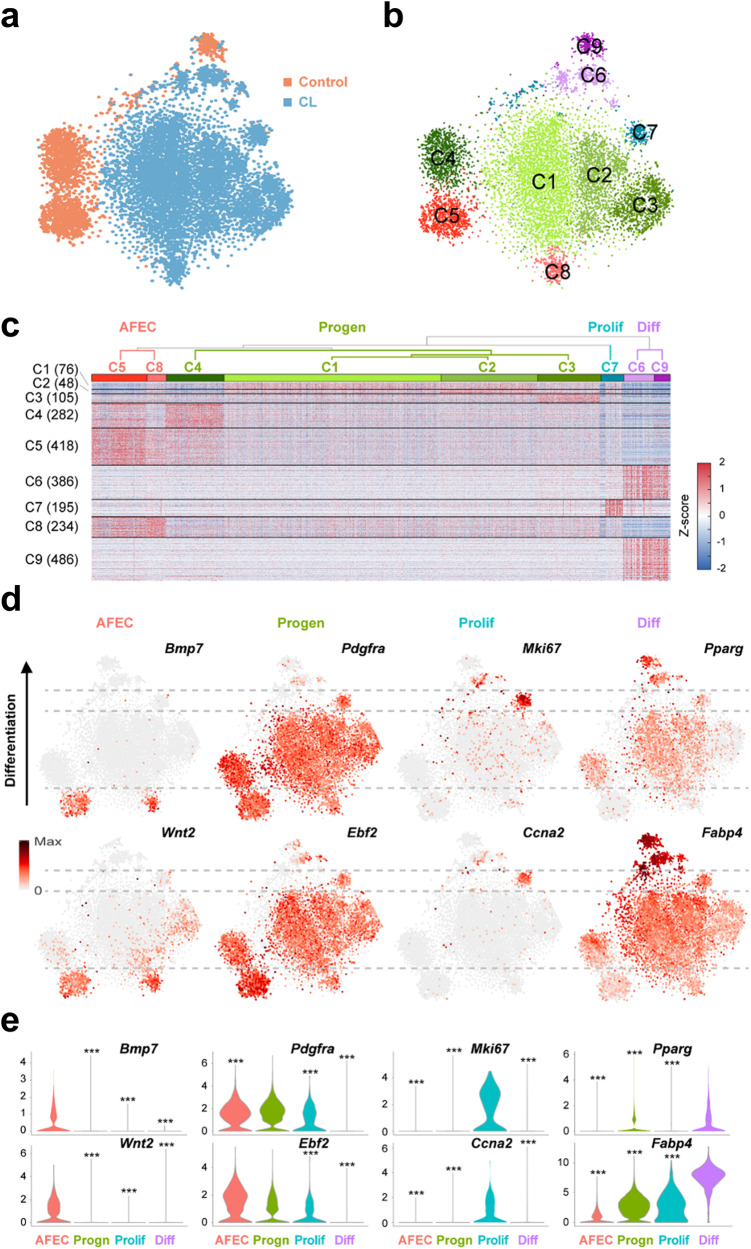
Cellular heterogeneity of control and CL-treated PDGFRA+ progenitors. **a** t-SNE plots of PDGFRA+ cells (dots) obtained from the control (orange, *n* = 3) and CL-treated (blue, *n* = 3) cells. **b** t-SNE plots showing the nine clusters (C1-9) of PDGFRA+ cells. Different colors were used to distinguish the clusters. **c** Four groups of the nine clusters (AFEC, Progen, Prolif, and Diff). A dendrogram of C1-9 was obtained from hierarchical clustering of the mean expression profiles in C1-9. The heatmap shows the increased (red) and decreased (blue) expression (Z score) of the upregulated genes (rows) in at least one cluster with respect to the median expression across all the samples (columns). The Z score was obtained by the inverse normal distribution of the mRNA expression level and log-normalized molecular count. The numbers of upregulated genes in each cluster are denoted in parentheses. **d** t-SNE plot showing the expression levels of the indicated representative upregulated genes in the four groups. The color bar represents the gradient of the mRNA expression level, and the same gradient was used for the range between the minimum (zero) and the maximum expression levels of each gene. **e** Violin plots showing the distributions of the mRNA expression levels of each representative upregulated gene in the four groups. ***P < 0.001 by one-way analysis of variance (ANOVA) with Tukey’s correction.

We then compared the transcriptomes of the selected control and CL-treated PDGFRA+ cells using t-distributed stochastic neighbor embedding (t-SNE). There was little overlap between the control and CL-treated PDGFRA+ cells, and CL treatment shifted the mRNA expression profiles of the PDGFRA+ cells (Fig. 2a). To examine the subpopulation structure of PDGFRA+ cells, we identified 9 major clusters (C1-9) of PDGFRA+ cells, varying from 310 to 4203 cells per cluster, using a graph-based clustering approach (Fig. 2b and Supplementary Fig. 2a). We then identified genes that defined these clusters by selecting the genes with significantly (*P* < 0.05) upregulated expression in each cluster compared to those in the other clusters (Supplementary Table 1). Interestingly, these genes exhibited shared upregulation in multiple clusters (C1-4, C5 and 8, and C6 and 9; Fig. 2c). Based on this observation, we further merged the 9 clusters into four groups (dendrograms of C1-9 in Fig. 2c) by clustering the mean expression profiles of the genes in the clusters (Supplementary Fig. 2b) and defined them as adipogenic factor-expressing cells (AFEC, C5 and 8), adipocyte progenitors (Progen, C1-4), and proliferating (Prolif, C7) and differentiating (Diff, C6 and 9) cells through the following analyses of the upregulated genes in each group.

Compared with those in the other groups, the cells in the AFEC group exhibited greater expression of proadipogenic factors, including bone morphogenetic proteins (*Bmp1*-*4* and *7*)^18^ and *Wnt2*^19^ (Fig. 2d, e and Supplementary Fig. 2c, d), suggesting that these cells may secrete adipogenic factors. Cells in the Progen group highly expressed progenitor markers, including *Pdgfra, Ebf2* and delta-like noncanonical Notch ligand 1 (*Dlk1*), which prevent adipocyte differentiation^20^. Interestingly, the expression of these markers was suppressed in the Diff group. These data suggest that these cells are resting preadipocytes. The Prolif group was strongly enriched in genes involved in the mitotic cell cycle, including the proliferation marker Ki-67 (*Mki67*), cyclin-dependent kinases and inhibitors (*Cdk1* and *Cdkn2c/2d/3*), cyclins (*Ccna2/b1/b2*), and cell division cycle associated (*Cdca3/a8/a20*), suggesting that these cells are actively proliferating. Cells in the Diff group exhibited high expression of genes involved in early adipogenesis, including peroxisome proliferator activated receptor gamma (*Pparg*), fatty acid binding proteins (*Fabp4/5*), carbonic anhydrase 3 (*Car3*), adiponectin receptor 1 (*Adipor1*), lipase (*Lipe*), and tissue inhibitor of matrix metalloproteinase 1 (*Timp1*). Consistently, the following processes related to adipogenic factor secretion, adipocyte progenitors, progenitor proliferation, and differentiation into adipocytes were significantly (*P* < 0.01) enriched in the upregulated genes in the AFEC, Progen, Prolif, and Diff groups (Supplementary Fig. 2e): ‘secretion by cell’ and ‘BMP signaling’ (AFEC); ‘extracellular matrix (ECM) organization’, ‘cell-matrix/substrate adhesion’ and ‘cell migration’ (Progen); ‘mitotic cell cycle’ and ‘cell division’ (Prolif); and ‘fat cell differentiation’ and ‘actin filament organization’ (Diff).

### PDGFRA+ cells expressing DPP4 have the characteristics of nonadipogenic niche cells

**Fig. 3:**
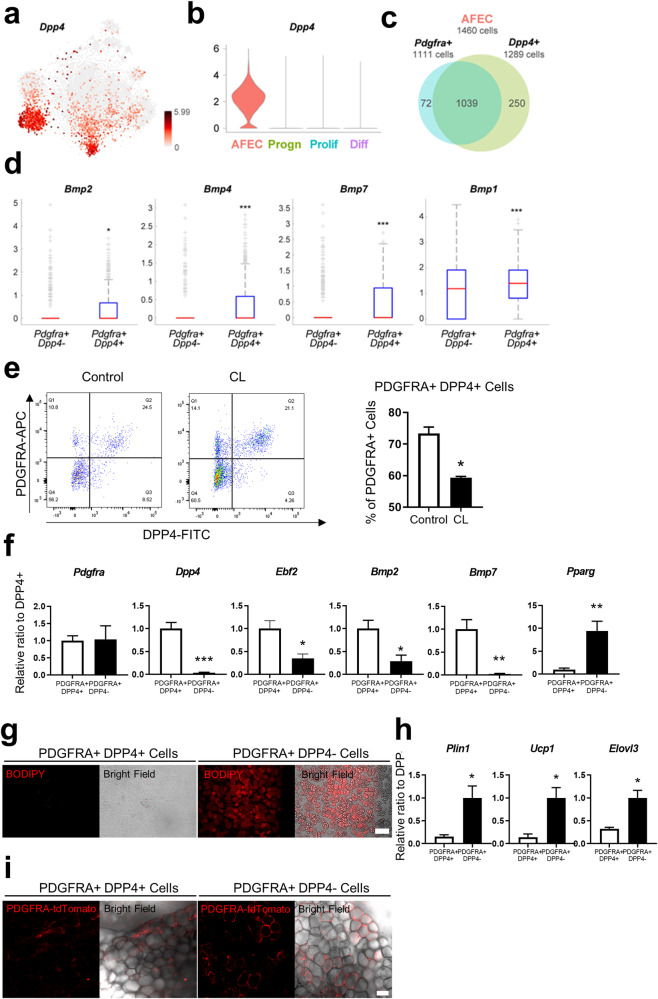
Non-adipogenic PDGFRA+ and DPP4*+* niche cells. **a** t-SNE plot showing the mRNA expression level of DPP4 across the cells in the four groups. The color bar represents the gradient of the mRNA expression level. **b** Violin plot showing the distributions of *Dpp4* mRNA expression in the indicated four groups. **c** Venn diagram showing the relationship between PDGFRA+ (1111 cells) and *Dpp4*+ cells (1289 cells) in the AFEC group (1460 cells). **d** Comparison of the mRNA expression levels of the indicated genes between *Pdgfra*+ *Dpp4*+ and *Pdgfra*+ *Dpp4*- cells. **e** Flow cytometric analysis of PDGFA and DPP4 expression in SVCs from the iWAT of control mice and mice treated with CL for 3 days. **f** Relative mRNA expression levels of the indicated genes in PDGFRA+ DPP4+ and PDGFRA+ DPP4− cells isolated from iWAT of mice with respect to the mean level in PDGFRA+ DPP4− cells. **g** Representative images of PDGFRA+ DPP4+ and PDGFRA+ DPP4− cells cultured under standard adipogenic conditions. Bar = 100 μm. **h** Relative mRNA expression levels of the indicated genes in PDGFRA+ DPP4+ and PDGFRA+ DPP4− cells cultured under standard adipogenic conditions. **i** Representative images of Matrigel gels containing PDGFRA+ DPP4+ and PDGFRA+ DPP4− cells 3 weeks after transplantation. Bar = 50 μm. The data were analyzed by an unpaired two-tailed *t* test (mean ± SEM; *n* = 3–5; *P < 0.05, **P < 0.01 and ***P < 0.001).

Adipocyte progenitors exist in various metastable states and can be induced toward adipogenic or nonadipogenic fates. Previous studies have suggested that subpopulations of PDGFRA+ progenitors exhibit different degrees of adipogenic potential^6,21^. Our data revealed two different groups of PDGFRA+ progenitors, AFECs and Progen cells, which share the expression of progenitor markers and showed upregulation of genes involved in progenitor-related processes (‘ECM organization, ‘cell-matrix/substrate/cell adhesion’, and ‘cell migration’; Supplementary Fig. 2e). In addition to these similarities, these groups also exhibited different molecular features. For example, the AFEC group showed higher expression of adipogenic factors (BMPs; Fig. 2d and Supplementary Fig. 2c) than did the Progen group, suggesting potential differences between the two groups in terms of the nature or strength of adipogenic potential. On the other hand, compared with the Prolif group, the AFEC group exhibited very low expression of cell proliferation markers (Fig. 2d and Supplementary Fig. 2c) but high expression of extracellular protease inhibitors (*Timp3* and *Pi16*; Supplementary Fig. 3a), which were downregulated during differentiation^7^, suggesting that the AFEC group is not actively proliferating or differentiating. To examine the adipogenic potential of the AFECs, we first evaluated the expression of a surface marker in the AFEC group. Among the upregulated genes in the AFEC group, 27 genes whose encoded proteins were localized in the plasma membrane according to the Gene Ontology (GO) cellular component analysis were identified. Then, we selected *Dpp4* as a marker that showed the strongest expression in the AFEC group compared to the other groups (Fig. 3a, b and Supplementary Fig. 3b), which is consistent with the findings of other recent scRNA-seq studies^7,8^.

Among the 1460 cells in the AFEC group, 1111 and 1289 cells expressed *Pdgfra* and *Dpp4*, respectively, and 93.5% of the *Pdgfra*-expressing cells also expressed *Dpp4* (Fig. 3c). Interestingly, these *Pdgfra*+ *Dpp4*+ cells had higher expression of *Bmp2*, *Bmp4*, *Bmp7*, and *Bmp1* than did the *Pdgfra*+ *Dpp4*− cells (Fig. 3d). Next, flow cytometric analysis showed that PDGFRA+ DPP4+ cells accounted for 73.4% and 59.3% of the PDGFRA+ cells in the control and CL-treated groups, respectively (Fig. 3e); these cells corresponded to C5 (control) and C8 (CL-treated), respectively, in the AFEC group **(**Fig. 2a, b). Quantitative real-time polymerase chain reaction (qRT–PCR) analysis confirmed that the mRNA expression levels of *Bmp2* and *Bmp7* were greater in PDGFRA+ DPP4+ cells than in PDGFRA+ DPP4− cells (Fig. 3f). FACS-sorted PDGFRA+ DPP4+ cells failed to differentiate into lipid+ adipocytes on standard adipogenic cocktail medium at 7 days after adipogenic induction, while PDGFRA+ DPP4− cells accumulated lipids (Fig. 3g). qRT‒PCR analysis further confirmed the absence of adipocyte markers, such as *Plin1, Ucp1 and Elovl3*, in PDGFRA+ DPP4+ cell cultures, indicating the lack of adipogenic potential of PDGFRA+ DPP4+ cells (Fig. 3h). Next, we transplanted Matrigel-containing PDGFRA+ DPP4+ cells or PDGFRA+ DPP4− cells isolated from PDGFRA-Cre/tdTomato mice. This transplantation assay confirmed the lack of adipogenic potential in PDGFRA+ DPP4+ cells compared to PDGFRA+ DPP4− cells (Fig. 3i). Collectively, these data suggest that PDGFRA+ DPP4+ cells are possibly nonadipogenic niche cells that produce and secrete adipogenic factors, thereby promoting the beige adipogenesis of neighboring cells.

In addition to their adipogenic potential, DPP4+ cells might serve as niche cells influencing adipogenesis. While we did not directly assess the specific roles of DPP4+ cells in adipogenesis, we investigated the effects of a DPP4 inhibitor on the adipogenic differentiation of C3H10T1/2 cells in vitro. We demonstrated that treatment with sitagliptin inhibited the adipogenic differentiation of C3H10T1/2 cells, as indicated by BODIPY and Oil Red O staining (Supplementary Fig. 5). This observation suggested that DPP4 activity may be required for adipogenic differentiation and that PDGFRA+ DPP4+ cells could serve as a source of DPP4.

### The BMP, Hedgehog, and Notch signaling pathways are serially activated during the differentiation of PDGFRA+ cells

**Fig. 4:**
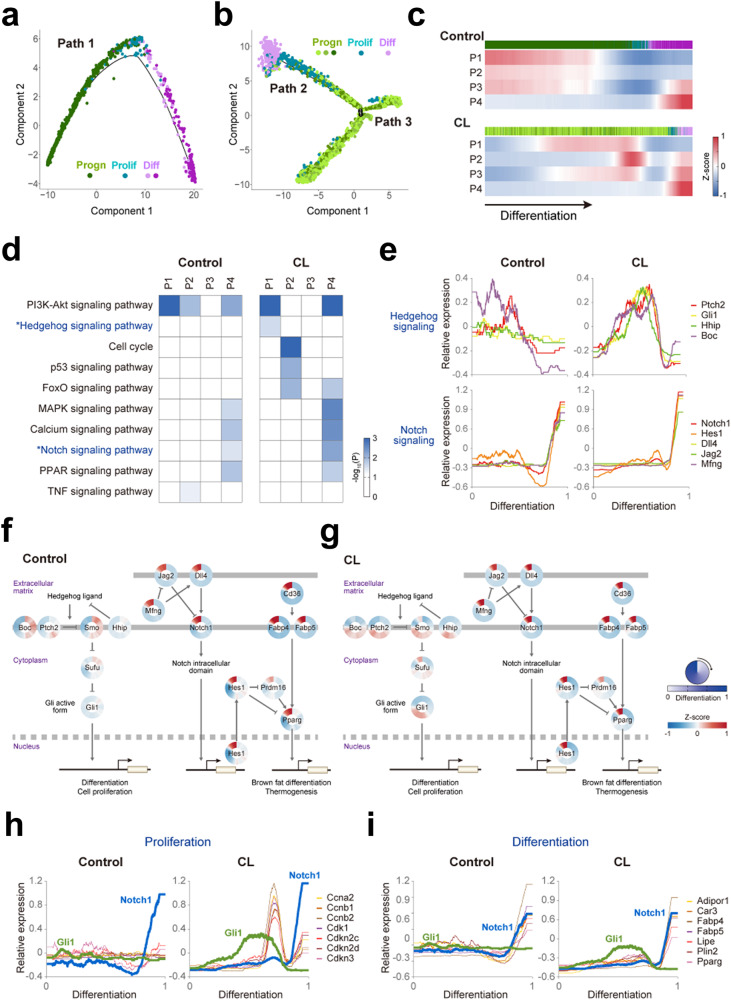
Serial activation of Hedgehog and Notch signaling during the de novo differentiation of PDGFRA+ cells. t-SNE plots showing the differentiation trajectories of control (**a**) and CL-treated (**b**) PDGFRA+ progenitors. Linear (Path 1) and bifurcating (Paths 2 and 3) trajectories were obtained for control and CL-treated PDGFRA+ progenitors. Different colors are used to distinguish the clusters in the Progen, Prolif, and Diff groups. **c** Four patterns (P1-4) showing the mean expression (Z score) of genes upregulated early, middle, or late along Path 1 (top) and Path 2 (bottom). The colored bars on the top of the heatmaps denote the cluster memberships of individual PDGFRA+ progenitors in the four groups. The pseudotime along the differentiation trajectory increases along the differentiation axis (arrow). **d** Signaling pathways enriched by the genes in P1-4 under control or CL treatment conditions. The heatmap shows the enrichment significance defined by –log_10_(*P*), where *P* is the enrichment *P* value from the EASE test in DAVID. The color bar represents the gradient of –log_10_(P). **e** Temporal relative expression profiles of the indicated representative genes involved in Hedgehog (top) and Notch signaling (bottom) during differentiation under control (left) and CL treatment (right) conditions. Relative expression profiles were obtained as described in the Materials and Methods section. Network models showing serial activation of Hedgehog and Notch signaling under control (**f**) and CL treatment conditions (**g**). The circular heatmap represents the increased (red) or decreased (blue) relative expression (Z score) of the indicated genes in the two pathways during differentiation (top legend). The color bar shows the gradient of the Z score. Arrows and suppression symbols represent activation and inhibition, respectively, of signaling reactions. The thick solid and dotted lines denote the plasma and nuclear membranes, respectively. The binding of translocalized transcription factors to the promoters of target genes is also shown. Temporal relative expression profiles of the indicated representative genes related to proliferation (**h**) and differentiation (**i**) under control (left) and CL treatment (right) conditions. The expression profiles of *Gli1* and *Notch1* are included as references.

Given the lack of adipogenic potential of the AFEC group, we next investigated the temporal order of the other three groups (Progen, Prolif, and Diff) during differentiation by reconstructing the differentiation trajectory of the control or CL-treated PDGFRA+ cells in the three groups. A single trajectory (Path 1) was identified for the control PDGFRA+ cells (Fig. 4a), whereas a bifurcating trajectory including Paths 2 and 3 was identified for the CL-treated PDGFRA+ cells (Fig. 4b). PDGFRA+ progenitors consistently underwent proliferation and subsequent differentiation into BAs under control conditions (Path 1) and CL treatment (Path 2). In contrast, PDGFRA+ progenitors in Path 3 were not capable of differentiating into BAs after proliferating. To determine the cellular signaling pathways that characterize differentiation along Paths 1 and 2, we next identified genes upregulated along each path and categorized them into four major patterns (P1-4): early, middle, or late upregulation along Path 1 or 2 (Fig. 4c and Supplementary Fig. 4a). The enrichment analysis revealed several signaling pathways significantly (*P* < 0.05) enriched in the genes in P1-4, including the PI3K-Akt, Hedgehog, p53, FoxO, MAPK, calcium, Notch, PPAR, and TNF signaling pathways (Fig. 4d). We also identified the genes upregulated along Path 3 and found that they were upregulated along Path 2, suggesting that they may contribute to proliferation but not to differentiation into BAs (Supplementary Fig. 4b).

Among the signaling pathways, we focused on the Hedgehog and Notch signaling pathways, which were enriched in early (P1 in Path 2) and late upregulated genes (P4 in Paths 1 and 2), respectively, suggesting that they underwent serial activation during differentiation (Fig. 4d). The enrichment was much greater under CL treatment than under control conditions. The other pathways were less likely to be associated with interrelationships among the Progen, Prolif, and Diff groups during differentiation because some of them (e.g., PI3K-Akt and FoxO) were enriched in multiple pathways with different kinetics or because the genes involved in these pathways were not the core components of the pathways. Moreover, pseudotime analysis of the Hedgehog (*Hhip*, *Ptch2*, *Boc*, and *Gli1*) and Notch (*Notch1*, *Dll4*, *Jag2*, *Hes1 and Mfng*) signaling pathway marker genes in the CL-treated cells showed that Hedgehog signaling activity increased at the early stage and then decreased after the peak in the middle stage, and Notch signaling activity began to increase at the late stage as Hedgehog signaling activity decreased (Fig. 4e and Supplementary Fig. 4c, d), suggesting that these genes underwent serial but reciprocal activation during differentiation. Network models of ligands, receptors, kinases, or transcription factors involved in Hedgehog and Notch signaling further confirmed the serial activation of these pathways during de novo differentiation (Fig. 4f, g). These dynamic transitions of the two pathways were also observed in the control condition, despite the decreased temporal synchronization of the Hedgehog signaling markers (Fig. 4e, f).

Interestingly, under CL-treated conditions, the expression of marker genes related to cell proliferation (*Cdk1*, *Cdkn2c/2d/3*, and *Ccna2/b1/b2*) began to increase as Gli1 expression began to decrease after the peak, indicating the reciprocal relationship between Hedgehog signaling and cell proliferation (Fig. 4h). This reciprocal expression pattern was not observed in the control condition. Notably, the expression of *Notch1* began to increase after CL treatment when Gli1 expression decreased to the basal level. In both the control and CL treatment conditions, the expression of marker genes of differentiated Bas (*Pparg*, *Fabp4/5*, *Car3*, *Adipor1*, *Plin2*, and *Lipe*) began to increase along with the induction of *Notch1* expression (Fig. 4i). Notch signaling has been shown to inhibit the de novo differentiation of adipocyte progenitors^22^. Thus, these data suggest that Notch signaling may be a negative modulator of the de novo differentiation of PDGFRA+ progenitors at the late stage. Taken together, these results indicate reciprocal functional interactions of Hedgehog signaling with Notch signaling and cell proliferation/BA differentiation.

### Dynamic interaction of Hedgehog signaling molecules during the differentiation trajectory is critical for the proliferation and differentiation of PDGFRA+ cells

**Fig. 5:**
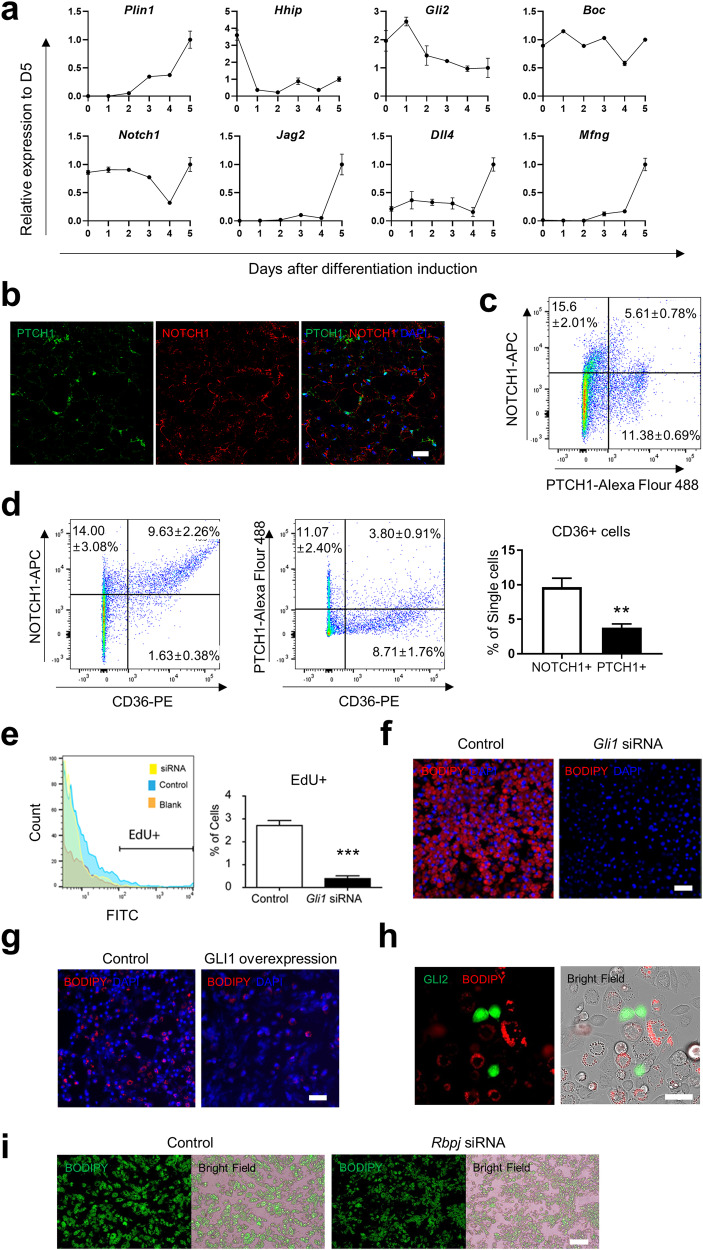
Dynamic interactions of Hedgehog signaling with the proliferation and differentiation of PDGFRA+ cells. **a** Relative mRNA expression levels of the indicated genes in C3H10T1/2 adipocytes after induction of differentiation with respect to the mean level on Day 5 (D5). **b** Representative immunostaining images showing PTCH1 and NOTCH1 expression in paraffin sections of iWAT from mice treated with CL for 3 days. Bar = 25 μm. **c** Flow cytometric analysis of PTCH1 and NOTCH1 expression in stromal vascular cells obtained from the iWAT of mice treated with CL for 3 days. **d** Flow cytometric analysis of CD36 expression in NOTCH1**+** and PTCH1+ cells. **e** Flow cytometric analysis of EdU incorporation in C3H10T1/2 cells treated with *Gli1* siRNA or control siRNA. **f** Representative images of C3H10T1/2 cells treated with *Gli1* siRNA or control siRNA and differentiated under standard adipogenic conditions. Bar = 100 μm. **g** Representative images of C3H10T1/2 cells overexpressing GLI1 and controls differentiated under standard adipogenic conditions. Bar = 100 μm. **h** C3H10T1/2 cells overexpressing GFP-fused GLI2 differentiated under standard adipogenic conditions. Bar = 50 μm. **i** Representative images of C3H10T1/2 cells treated with *Rbpj* siRNA or control cells differentiated under standard adipogenic conditions. Bar = 100 μm. The data were analyzed by an unpaired two-tailed t test (mean ± SEM, *n* = 4; **P < 0.01 and ***P < 0.001).

To test whether this reciprocal relationship between Hedgehog and Notch signaling is consistently observed during adipogenic differentiation, we next measured the mRNA expression levels of the representative marker genes for Hedgehog (*Hhip, Gli2* and *Boc*) and Notch (*Notch1, Jag2, Dll4*, and *Mfng*) signaling during in vitro adipogenic differentiation of C3H10T1/2 cells. The mRNA levels of *Hhip* and *Gli2* decreased during differentiation, while those of *Dll4, Jag2, Mfng*, and *Plin1* increased (Fig. 5a). The luciferase reporter system also demonstrated the reciprocal relationship between Hedgehog and Notch signaling during adipogenic differentiation (Supplementary Fig. 6). We then further examined the in vivo expression patterns of the Hedgehog (PTCH1) and Notch (NOTCH1) signaling proteins in iWAT isolated from C57BL/6 mice 3 days after CL treatment. Immunohistochemistry analysis confirmed the mutually exclusive expression of the two marker proteins for Hedgehog and Notch signaling in stromal vascular cells (SVCs) (Fig. 5b). Moreover, we examined the relationships of CD36-expressing cells with cells expressing NOTCH1 or PTCH1 under the same conditions. NOTCH1 was expressed in CD36+ early differentiating adipocyte precursors, while PTCH1 was expressed in CD36- undifferentiated cells (Fig. 5c, d). These data collectively support the reciprocal relationship between Hedgehog signaling and Notch signaling during both in vitro and in vivo adipogenic differentiation.

To investigate whether this reciprocal expression pattern of the two signaling pathways has functional implications for adipogenesis, we first examined the effect of Hedgehog signaling on cell proliferation by measuring the change in the number of EdU+ cells during growth medium culture after *Gli1* was knocked down in C3H10T1/2 cells. The proportion of EdU+ cells was significantly (*P* < 0.01) decreased by *Gli1* knockdown (Fig. 5e). Moreover, lipid staining revealed virtually no adipogenesis after *Gli1* knockdown or GLI1 overexpression, in contrast to the substantial amount of lipids synthesized without genetic manipulation of *Gli1* expression (Fig. 5f, g). Correspondingly, when GLI2 was overexpressed in C3H10T1/2 cells (green in Fig. 5h), no lipid accumulation (red in Fig. 5h) was observed on Day 7. These data collectively suggest that Hedgehog signaling has negative effects on both proliferation and differentiation. To examine the effect of Notch signaling on adipogenic differentiation, we next knocked down *Rbpj* in C3H10T1/2 cells and measured lipid levels on Day 7. Compared with scrambled siRNA treatment, *Rbpj* knockdown did not induce a significant difference in lipid content (Fig. 5i). Taken together, these data suggest that Hedgehog signaling is activated at the early stage of adipogenic differentiation and then suppressed at the late stage to induce differentiation, consistent with our findings from the trajectory analysis of the scRNA-seq data.

According to our single-cell analysis, AFECs were the major cell type expressing *Dpp4*. To assess the impact of Hedgehog or Notch signaling on *Dpp4* expression, we established a luciferase reporter system containing the *Dpp4* promoter sequence in HEK293T cells. Neither the genetic overexpression of GLI1 or NICD1 nor the use of pharmacological inhibitors (the Hedgehog inhibitor GANT61 or the indirect Notch inhibitor DAPT) altered *Dpp4* promoter activity (Supplementary Fig. 7). These findings indicate that Hedgehog and Notch signaling had no significant effects on *Dpp4* expression.

### Lineage tracing revealed that DPP4+ cells become adipocytes during hyperplastic expansion of iWAT induced by HFD feeding

**Fig. 6:**
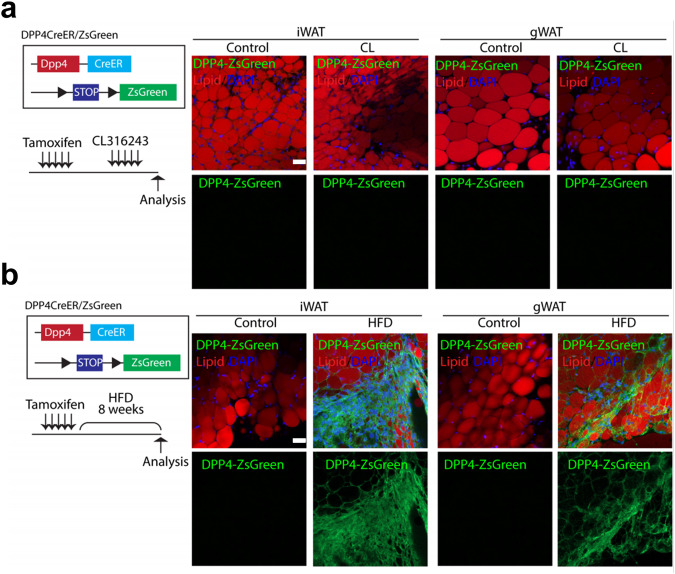
Genetic lineage tracing of DPP4+ cells in white adipose tissue. Representative images of adipose tissue stained with LipidTox obtained from DPP4-CreER/ZsGreen mice after 5 days of CL treatment (**a**) or 8 weeks of high-fat diet feeding (**b**). Bar = 50 μm.

We showed above that the isolated PDGFRA+ DPP4+ cells did not differentiate into BAs following CL treatment in vitro or after transplantation (Fig. 3). However, these findings appear to contradict previous findings that DPP4+ cells can undergo adipogenic differentiation. Our in vitro findings may no longer be valid during in vivo adipogenesis. We thus investigated whether DPP4+ cells become adipocytes in vivo through genetic tracing in DPP4-CreER/ZsGreen mice. Both iWAT and gWAT from DPP4-CreER/ZsGreen mice showed no change in DPP4 expression in response to CL treatment, as indicated by the lack of adipocytes with ZsGreen expression (Fig. 6a). These data indicate that no DPP4+ cells gave rise to adipocytes during beige adipogenesis induced by CL treatment, consistent with our in vitro findings.

Alternatively, we next investigated whether DPP4+ cells could differentiate into adipocytes under pathological conditions. A high-fat diet (HFD) is known to induce de novo adipogenesis in adipose tissue^8^. We thus hypothesized that DPP4+ cells might contribute to adipogenesis during HFD feeding. Thus, using tamoxifen-inducible DPP4-CreER/ZsGreen mice, we traced newly generated adipocytes from DPP4+ cells during 8 weeks of HFD feeding (Fig. 6b). Surprisingly, HFD feeding indeed induced adipogenic differentiation of DPP4+ cells in iWAT. Taken together, our results demonstrate the context-dependent adipogenic potential of DPP4+ cells, possibly accounting for the discrepancy between our findings and previous findings. In other words, while DPP4+ cells undergo no adipogenic differentiation during normal development, DPP4+ cells undergo adipogenic differentiation under HFD conditions, thereby contributing to hyperplastic expansion of adipose tissue during diet-induced obesity.

## Discussion

Our scRNA-seq analysis aimed to characterize the heterogeneity of PDGFRA+ cells that contribute to beige adipogenesis during the catabolic remodeling of adipose tissue induced by beta3 adrenergic stimulation. Notably, although we utilized a selective beta3 adrenergic receptor agonist to induce brown adipogenesis, other subtypes of beta-adrenergic receptors may contribute to the activation, recruitment, and thermogenesis of human brown adipocytes. Notably, Blondin et al.^23^ demonstrated that human brown adipocyte thermogenesis is modulated by the beta2 adrenergic receptor agonist formoterol rather than the beta3 adrenergic receptor agonist mirabegron. In contrast, Cero et al.^24^ showed that the beta3 adrenergic receptor regulates human brown/beige adipocyte lipolysis and thermogenesis through genetic silencing of beta3 adrenergic receptor expression and pharmacological agents in primary human adipocytes. However, further studies are needed to validate the applicability of these findings from beta3 adrenergic receptor stimulation-induced de novo brown adipogenesis models in a clinical setting.

While stem cells are known to require niche cells that support proliferation and differentiation^25^, the nature of these niche cells that support in vivo beige adipogenesis remains unclear. In this study, scRNA-seq analysis revealed that the AFEC group highly expressed BMPs and WNTs as a potential population of niche cells. Analysis of the upregulated genes in the AFEC group identified DPP4 as a surface marker that characterizes the AFEC group. We then experimentally demonstrated that PDGFRA+ DPP4+ cells isolated from iWAT had no adipogenic potential during in vitro adipogenic differentiation or after in vivo transplantation. More importantly, our genetic lineage tracing study indicated that virtually no DPP4+ cells became adipocytes in iWAT during beige adipogenesis, whereas HFD feeding induced the differentiation of DPP4+ cells to adipocytes, which may suggest the pathologic role of DPP4+ cell lineages in the hyperplastic expansion of WAT during obesity.

The DPP4+ cells identified in this study appear to have overlapping molecular phenotypes with the adipocyte stem cells defined by previous scRNA-seq studies^7,8^. Previous studies by Burl et al.^7^ identified heterogeneous cell types in adipose tissue responding to CL treatment, and the present study aimed to provide a comprehensive characterization of the cellular and molecular heterogeneity of PDGFRA+ progenitors responsible for CL-induced beige adipogenesis in iWAT. This approach allows us to delineate the signaling pathways involved in the sequential differentiation of beige adipocytes. Conversely, Merrick et al.^8^ reported that DPP4+ cells are multipotent mesenchymal progenitors that can give rise to more committed adipocyte progenitors and eventually mature adipocytes both in vitro and after in vivo transplantation. Although the adipogenic potential of mesenchymal stem cells has been demonstrated under artificial culture conditions, genetic tracing is necessary to determine the in vivo fate of DPP4+ cell lineages. Elucidation of the contribution of DPP4+ cells to adipose tissue remodeling induced by various stimuli would be beneficial.

Although we used DPP4 as a marker for AFECs, DPP4 is known to play an important role in the pathogenesis of type 2 diabetes. Functionally, DPP4 is a serine exopeptidase involved in the degradation of endogenous glucagon-like peptide-1 (GLP-1), a peptide hormone that decreases blood glucose levels by promoting insulin secretion. Therefore, DPP4 inhibitors have been used as antihyperglycemic/antidiabetic therapeutics. Knockout mouse studies have suggested that DPP4 inhibition has beneficial effects on metabolic diseases. For example, hepatocyte-derived DPP4 promotes visceral adipose tissue inflammation and insulin resistance in individuals with obesity^26^, and deletion of adipocyte-specific DPP4 expression protects against obesity-related metabolic dysfunction^27^. However, the systemic contribution of DPP4 derived from PDGFRA+ cells has not been investigated. Investigating the pathophysiological roles of PDGFRA+ DPP4- nonadipogenic niche cells (AFECs) in obesity-induced insulin resistance and metabolic dysfunction would be worthwhile. In this regard, detailed functional experiments are needed to determine the molecular mechanisms involved in modulating the adipogenic fate of AFECs in iWAT under both physiological and pathological conditions.

We identified several signaling pathways (PI3K-Akt, Hedgehog, p53, FoxO, MAPK, calcium, Notch, PPAR, and TNF signaling) enriched by genes showing early, middle, or late upregulation during the differentiation of PDGFRA+ adipocyte progenitors to BAs. Among these pathways, we focused on the Hedgehog and Notch signaling pathways because of their serial activation during differentiation. PI3K-Akt signaling was enriched by both early and late upregulated genes following CL treatment. FoxO signaling was enriched in both middle- and late-stage upregulated genes. These enrichment patterns suggest that these pathways may not be linked to the serial activation of the Progen, Prolif, or Diff groups. Additionally, the genes involved in the p53 and TNF signaling pathways included no core upstream or downstream factors but included those that could also be involved in other signaling pathways (e.g., cell cycle regulators in p53 signaling), suggesting potential false positives from the enrichment analysis of signaling pathways. Finally, the MAPK, calcium, and PPAR signaling pathways were reported to be associated with beige adipogenesis^28^. Like those involved in Notch signaling, these pathways are activated late during the course of differentiation. Among them, we selected Notch signaling, which had the largest number of core regulators that exhibited late activation patterns: *Notch1*, *Dll4*, *Jag2*, *Mfng*, and *Hes1* for Notch signaling; *Cacna1c* and *Calm1* for calcium signaling; *Mapk3* and *Map3k20* for MAPK signaling; and *Pparg*, *Cd36*, and *Fabp4/5* for PPAR signaling. Nevertheless, functional experiments can be designed to investigate the functional interactions of Hedgehog signaling with MAPK, calcium, and PPAR signaling during differentiation, as can be done for Notch signaling.

Our study demonstrated the power of scRNA-seq for characterizing the core signaling pathways underlying beige adipogenesis, given its cellular heterogeneity, compared to bulk RNA-seq. However, whether Hedgehog signaling promotes or inhibits the differentiation of progenitors into brown or beige adipocytes has been controversial^29,30^. The differentiation trajectory analysis showed that Hedgehog signaling was induced and then suppressed during the de novo differentiation of PDGFRA+ progenitors in iWAT. In the suppression phase, the expression of markers of cell proliferation and differentiation was induced as Hedgehog signaling activity decreased, suggesting that Hedgehog signaling negatively regulates de novo differentiation. Prior to the suppression phase, however, progenitors cannot initiate differentiation without the induction of Hedgehog signaling, indicating that Hedgehog signaling positively regulates de novo differentiation. Overall, Hedgehog signaling can promote or suppress the de novo differentiation of a progenitor, depending on whether the cellular state of the progenitor is in the induction or suppression phase. In contrast, given the cellular heterogeneity, bulk RNA-seq can reveal the molecular status of progenitors in the induction and suppression phases, thereby revealing the dynamic transiting characteristics of Hedgehog signaling. However, the factors that drive the initial induction of Hedgehog signaling remain unclear. According to our scRNA-seq analysis, the expression of BMPs upregulated in the AFEC group began to decrease in the progenitor group during differentiation. Previously, Hedgehog signaling activity in the ventral neural tube was shown to be regulated by secreted BMPs or their inhibitors^31^. These data suggest that BMPs secreted from PDGFRA+ DPP4+ niche cells in the AFEC group may contribute to the initial induction of Hedgehog signaling.

In conclusion, our scRNA-seq analysis enabled the categorization of PDGFRA+ cells into AFEC, Progen, Prolif, and Diff groups. PDGFRA+ DPP4+ cells in the AFEC group were characterized as a potential niche cell population that secretes proadipogenic BMPs. Trajectory analysis revealed that serial activation of Hedgehog and Notch signaling could be critical for the coordinated regulation of proliferation and differentiation of PDGFRA+ progenitors during de novo differentiation in iWAT. Bulk RNA-seq analysis would not have effectively categorized PDGFRA+ cells into four cell groups due to the mixed molecular signatures of these groups. Moreover, these findings could lead to potential therapeutic strategies for metabolic disorders, such as obesity and type 2 diabetes mellitus, for which metabolic benefits can be obtained through the manipulation of beige adipogenesis in iWAT. For example, the induction or suppression of Hedgehog signaling in PDGFRA+ cells can be modulated to control the dynamics of beige adipogenesis during pathological remodeling of adipose tissue in iWAT. Moreover, PDGFRA+ DPP4+ niche cells can be targeted to modulate the initiation and progression of adipogenic differentiation of PDGFRA+ progenitors in iWAT.

## Supplementary information

Supplementary Figures and Tables

Supplementary Table 1

## Data Availability

All the data generated or analyzed during this study are included in this article and its supplementary information files or are available from the corresponding authors upon request. The scRNA-seq data were deposited in the SRA database (accession ID: PRJNA523501/SRP187338).
