# Supplementary material for: Single-cell transcriptomic analysis reveals dynamic activation of cellular signaling pathways regulating beige adipogenesis
Source: Exp Mol Med. 2024 Oct 28;56(10):2309–22. doi: 10.1038/s12276-024-01252-9 (PMC11541910; doi:10.1038/s12276-024-01252-9)
Supplement: Supplementary file 1 — Supplementary Figures and Tables [file 12276_2024_1252_MOESM1_ESM.pdf]

Single-cell transcriptomic analysis reveals dynamic activation of cellular signaling pathways regulating beige adipogenesis

This file includes Supplementary Figures 1-7 and Supplementary Table 1-2.

Supplementary Figures

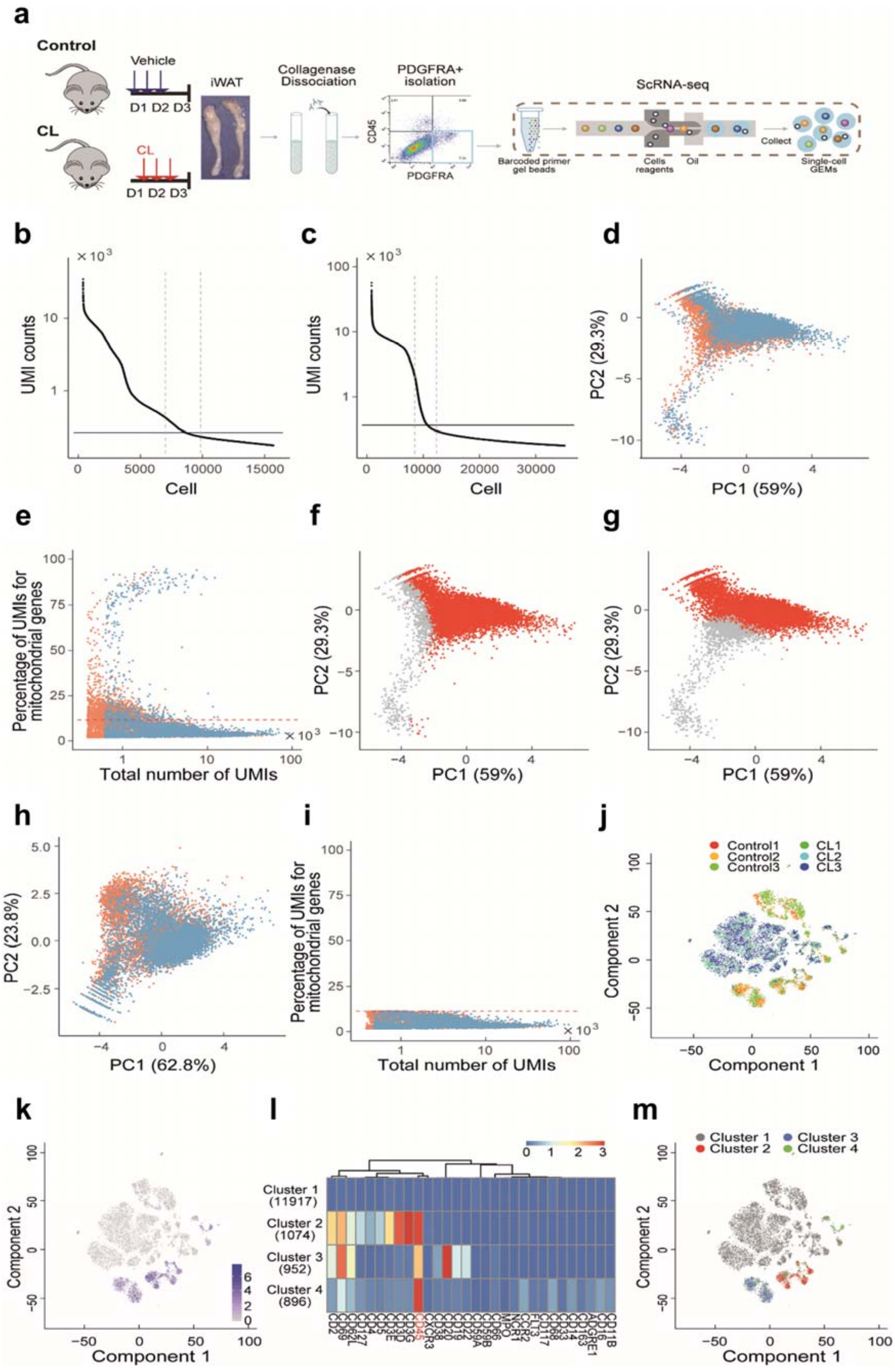

Supplementary Fig. 1: Selection of PDGFRA+ cells for the analysis of cellular heterogeneity.

a Overall scheme for scRNA-seq analysis of PDGFRA+ cells isolated from inguinal white adipose tissue (iWAT). Isolation of PDGFRA+ cells and generation of single cell GEMs and sequencing libraries were separately performed for independent triplicates of controls (n = 3) and CL-treated mice (n = 3), and the pooled library was then sequenced. **b-c** Cell barcode rank plots. Barcodes of control (**b**) or CL-treated PDGFRA+ cells (**c**) are ordered by the number of UMIs in a descending manner and the log-transformed UMI counts are plotted. A knee point used to determine the number of cells detected is indicated by the horizontal solid line, and the left and right 1,000 cells from the knee point used to determine the knee point are indicated by the dotted lines (Materials and Methods). **d** 2-dimensional PCA score plot showing control (orange) or CL-treated PDGFRA+ cells (blue) after PCA was applied to QC metrics of control and CL-treated cells (dots). The percentages of the explained variances by the first two principal components (PC1 and PC2) are shown in the parenthesis. **e** A scatter plot of the percentage of UMI counts assigned to mitochondrial encoded genes versus the total number of UMIs per cell barcode. **f-g** PCA score plot showing cells of good quality (red dots) with the percentage of genes with expression values of zero (dropout) less than 99.5% (**f**) and the percentage of UMI counts assigned to mitochondrial encoded genes less than 10% (**g**). **h-i** PCA score plot (**h**) and the scatter plot of mitochondrial UMI counts versus the total number of UMIs (**i**) obtained after discarding cells of poor quality. **j** t-SNE plots of all cells of good quality from

three independent control (Control 1-3) and CL-treated samples (CL1-3) after batch effect removal. The homogeneous mix of cells from the three replicates under control or CL-treated condition indicates that batch effect was effectively removed. **k** t-SNE plot showing expression levels of CD45, an immune cell marker, in individual cells. The color bars represent the gradient of log-normalized molecular count. **l** Four clusters of cells identified through k-means clustering (Materials and Methods) using expression levels of hematopoietic markers. Clusters 2-4 included the CD45-positive immune cells shown in **k**. **m** t-SNE plot of CD45-positive PDGFRA+ cells in Cluster 1 (gray) and CD45-positive immune cells in Clusters 2-4 (red, blue, and green, respectively). PDGFRA+ cells in Cluster 1 were then used for the analysis of cellular heterogeneity (**Fig. 2**).

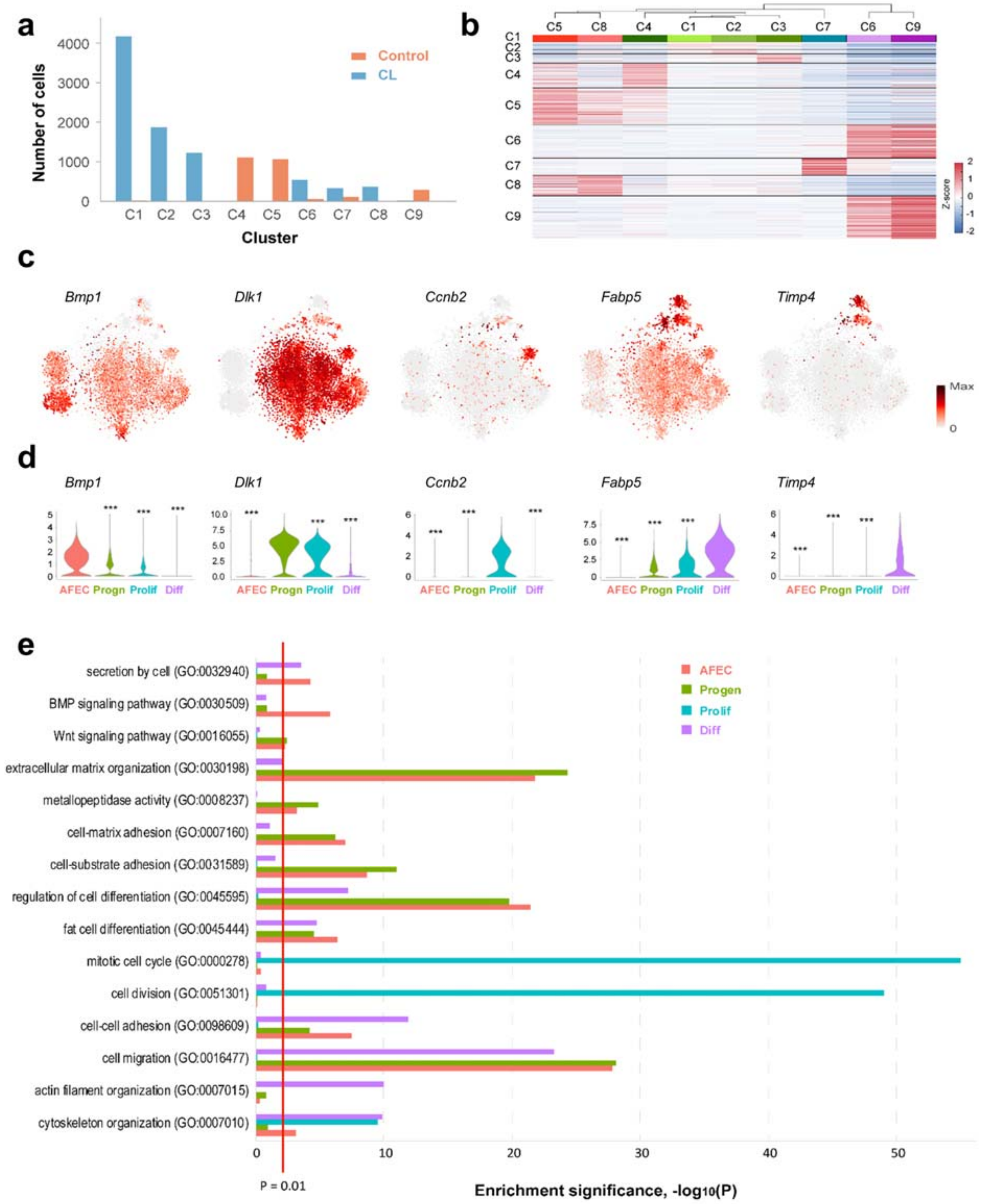

Supplementary Fig. 2: Four major clusters of PDGFRA+ cells.

a Numbers of control (blue) and CL-treated (orange) PDGFRA+ cells in the 9 major clusters (C1-9). **b** Clustering of the mean expression profiles of the genes (**Fig. 2c**) defining the 9 major

clusters (Euclidian distance as a dissimilarity measure and complete linkage). The dendrogram shows the agglomerative grouping of C1-9. For each gene, the mean expression profile was obtained as the average of Z scores (Materials and Methods) of the cells in the corresponding cluster. The color bar represents the gradient of Z score. **c** t-SNE plot showing the expression level of the following representative up-regulated genes in four groups of the 9 major clusters (**Fig. 2c**): *Bmp1* for the adipogenic factor expressing cell (AFEC) group; *Dlk1* for the progenitor (Progen) cell group; *Ccnb2* for the proliferating (Prolif) cell group; and *Fabp5* and *Timp4* for the differentiating (Diff) cell group. The color bar represents the gradient of mRNA expression level, and the same gradient was used for the range between the minimum (zero) and the maximum expression levels of each gene. **d** Violin plot showing distributions of mRNA expression levels of each representative up-regulated gene (**c**) in the four groups. **e** Cellular processes enriched by the up-regulated genes in the four groups (AFEC, Progen, Prolif, and Diff). The enrichment significance is shown as $-\log_{10}(P)$ where P is the enrichment P-value from EASE test in DAVID. The red line indicates a cutoff of the enrichment P-value ($P = 0.01$).

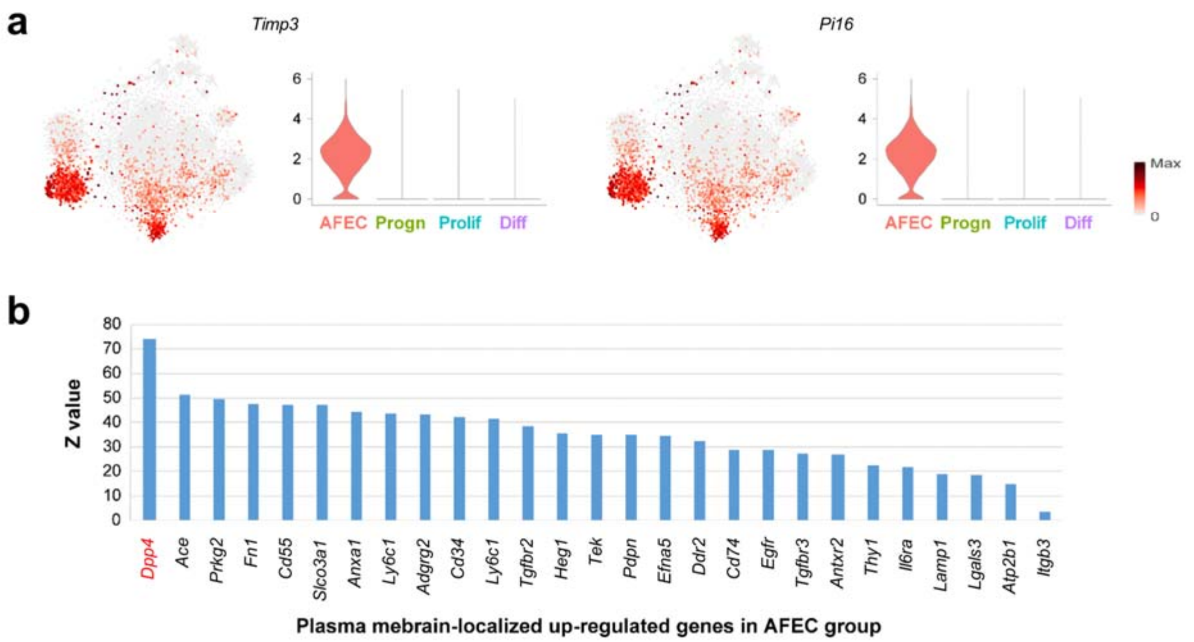

Supplementary Fig. 3: Selection of a surface marker for AFEC group.

a t-SNE plot showing the expression level of *Timp3* and *Pi16* up-regulated in AFEC group and violin plot showing distributions of mRNA expression levels of *Timp3* and *Pi16* in the four groups. In t-SNE plot, the color bar represents the gradient of mRNA expression level, and the same gradient was used for the range between the minimum (zero) and the maximum expression levels of each gene. **b** Z value representing the relative ratio of expression levels of the 27 plasma membrane-localized genes in AFEC group to the other groups. The selected *Dpp4* is denoted in red.

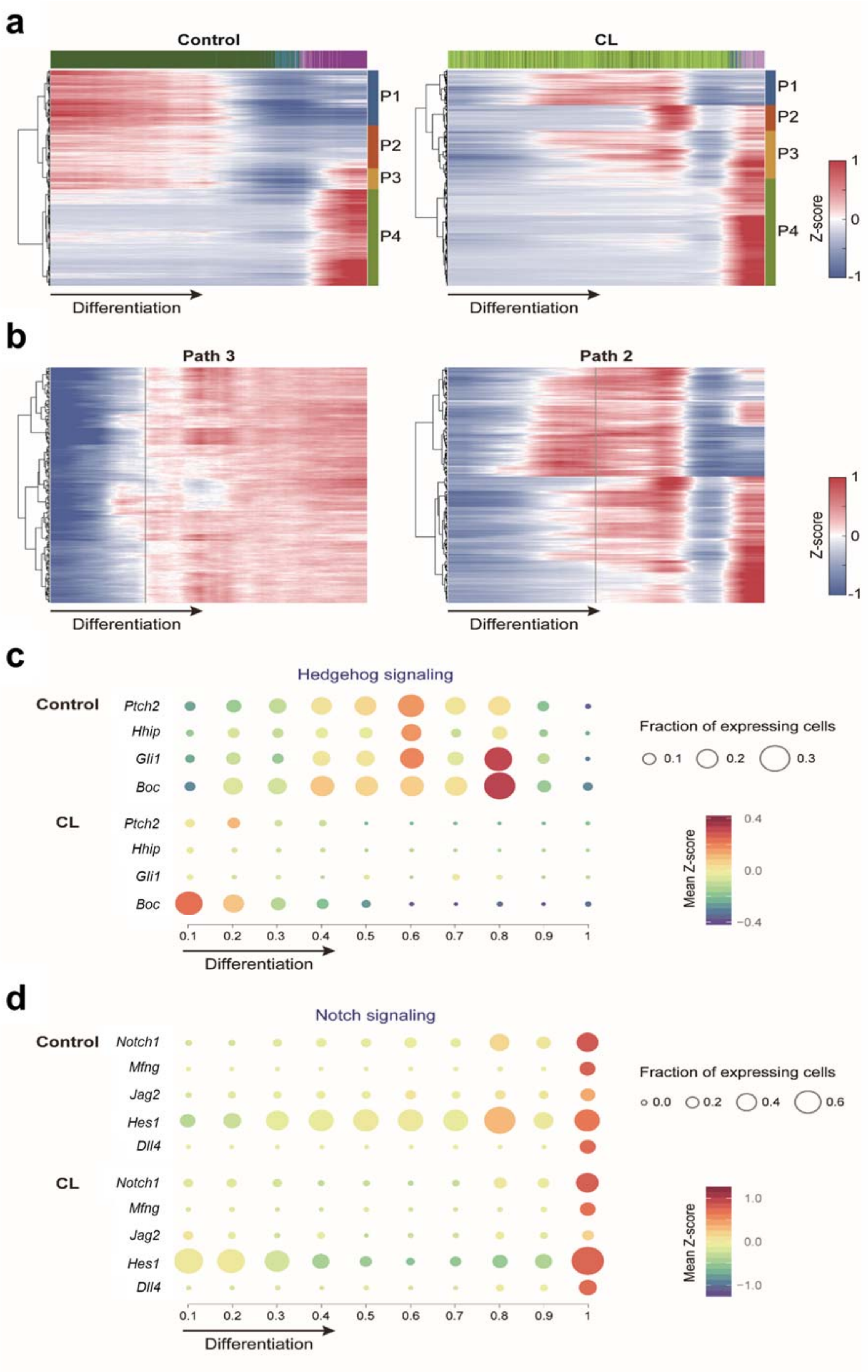

a
Control
CL
P1
P2
P3
P4
Z-score
1
0
-1
Differentiation
b
Path 3
Path 2
c
Hedgehog signaling
Control
CL
Ptch2
Hhip
Gli1
Boc
Fraction of expressing cells
0.1
0.2
0.3
Mean Z-score
0.4
0.0
−0.4
0.1 0.2 0.3 0.4 0.5 0.6 0.7 0.8 0.9 1
d
Notch signaling
Notch1
Mfng
Jag2
Hes1
Dll4
0.0
0.2
0.4
0.6
1.0
−1.0

Supplementary Fig. 4: Serial activation of Hedgehog and Notch signaling along the *de novo* differentiation of PDGFRA+ cells.

a Heat maps showing the expression (Z-score) profiles of early, middle, or late up-regulated genes in four patterns (P1-4) along Path 1 (left) and Path 2 (right). Colored bars on the top of heat maps denote cluster memberships of individual PDGFRA+ progenitors in the four groups. Pseudotime along the differentiation trajectory increases along the differentiation axis (arrow). The color bar denotes the gradient of Z-score. **b** Heat map showing the expression (Z-score) profiles of up-regulated genes along Path 3 (left). Expression profiles of these genes along Path 2 are also shown in the right heat map. Gray lines indicate the bifurcation point. **c-d** Dot plots showing the mean expression levels of the representative genes in Hedgehog (*Ptch2*, *Gli1*, *Hhip*, and *Boc*; **c**) and Notch signaling (*Notch1*, *Hes1*, *Dll4*, *Jag2*, and *Mfng*; **d**) at 10 time intervals along Path 1 (top, Control) and Path 2 (bottom, CL). The color bar represents the gradient of the mean expression levels (Z-score). In each time interval, the fraction of cells expression each representative gene is denoted by the circle size (see the legend).

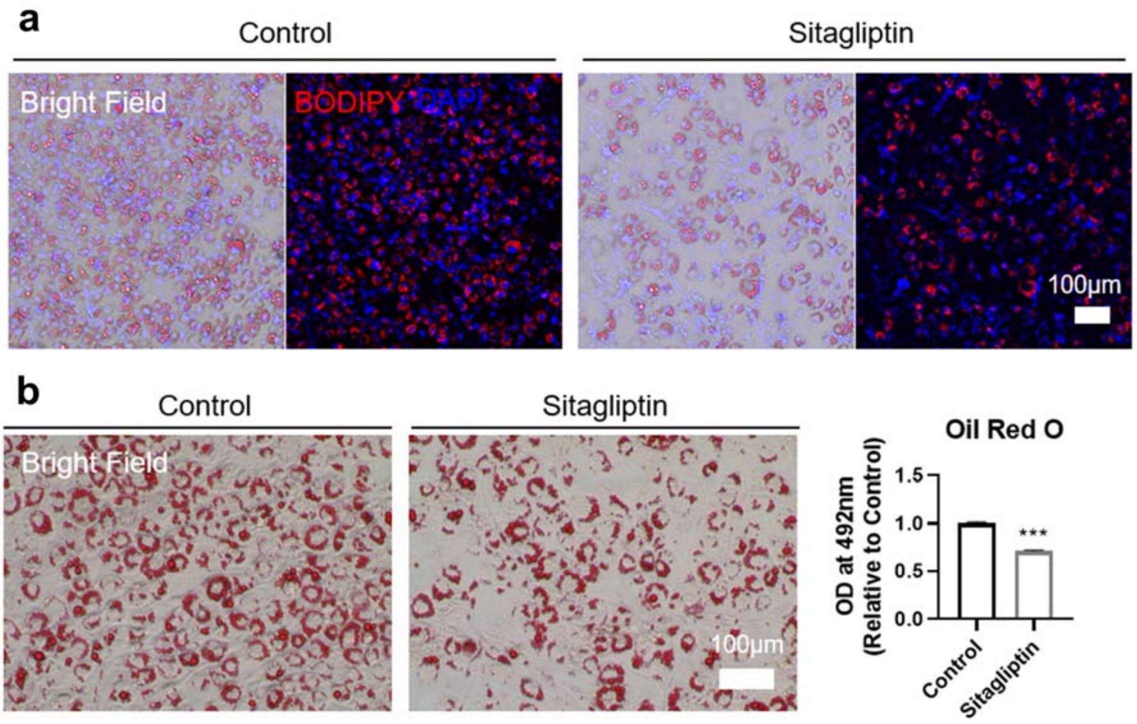

Supplementary Fig. 5: Effects of sitagliptin on adipogenic differentiation of C3H10T1/2 cells.

Representative images of neutral lipid staining by BODIPY (**a**) and Oil Red O (**b**) in C3H10T1/2 adipocytes treated with 100 μM of sitagliptin during the differentiation process. Data were analyzed using an unpaired, two-tailed t-test (n = 4, mean ± SEM; ***$P < 0.001$).

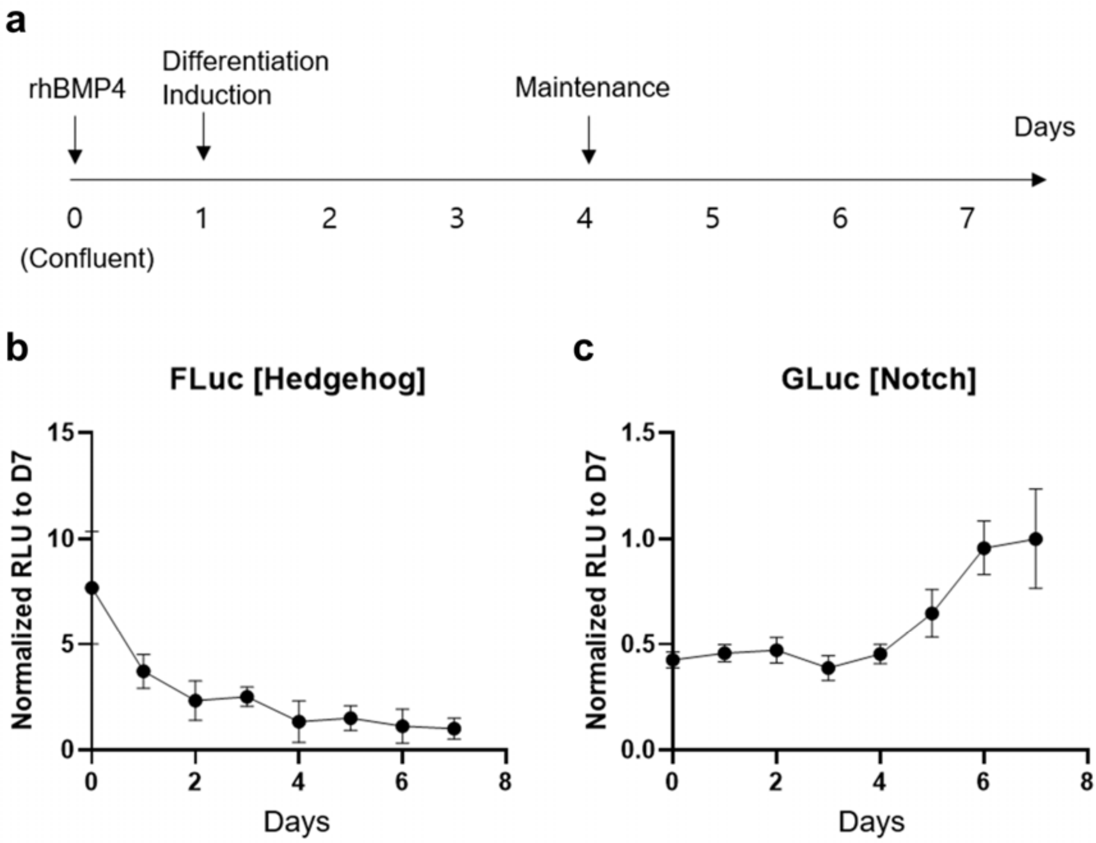

Supplementary Fig. 6: Luciferase reporter assay of Hedgehog and Notch signaling activation during adipogenic differentiation of C3H10T1/2 cells.

As described in the Materials and Methods section, the promoter containing multiple GLI binding sites responds to Hedgehog signaling and expresses firefly luciferase (FLuc). The CBF promoter responds to Notch signaling and expresses Gaussia luciferase (GLuc), which is secreted into the media, enabling simultaneous monitoring of Hedgehog and Notch signaling. **a** Schematic diagram illustrating the differentiation process of C3H10T1/2 cells. **b-c** FLuc (**b**) and GLuc (**c**) reporter activity of differentiating C3H10T1/2 cells (n = 6, mean ± SEM).

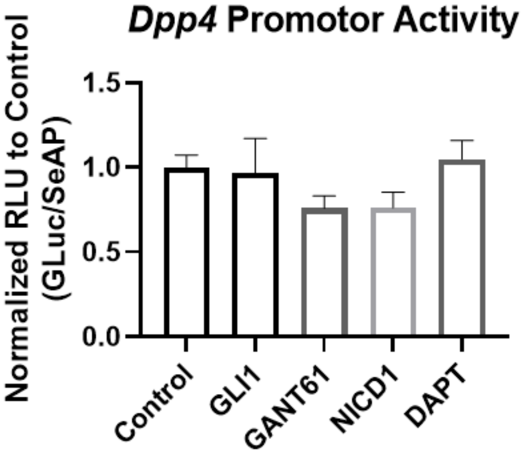

Supplementary Fig. 7: *Dpp4* promoter luciferase assay in HEK293T cells.

HEK293T cells were transfected with a luciferase plasmid containing *Dpp4* promoter sequence. To activate Hedgehog and Notch signaling, GLI1 (Addgene plasmid #62967) or NICD1 (Addgene plasmid #44471) expression plasmids were co-transfected, respectively. For pharmacological inhibition, at 24 h post-transfection, GANT61 (10 μM) or DAPT (10 μM) were treated for 24 hours to suppress Hedgehog and Notch signaling, respectively. Data were analyzed by an unpaired, two-tailed t-test (n = 4, mean ± SEM).

Supplementary Tables

Supplementary Table 1. List of differentially expressed genes characterizing cell clusters.

See Excel file entitled “Supplementary Table 1.xlsx.”

Supplementary Table 2. List of primers used for real time-quantitative polymerase chain reactions.

Genes	Forward (5′→3′)	Reverse (5′→3′)
Pdgfra	TGTGCCCATTCGCAGGAAGAG	TTGGCCACCTTGACACTGCG
Dpp4	TGTGATGTGGTGTGGGCTAC	AGGTGAAGTGAGGTTCTGCG
Ebf2	ACAGCCTTCGTGGACTTTGT	GCTATGGGCTGTTTGGTGAC
Bmp2	CTCTCTCAATGGACGTGCCC	AACACTAGAAGACAGCGGGTC
Bmp7	GGCCTGCAAGAAACATGAGC	AGTGAACCAGTGTCTGGACG
Pparg	AGCTGAATCACCCAGAGTCC	GGGTGAAGGCTCATGTCTGT
Plin1	GAGTCAGCGACAGCTTCTTC	CTTGACGAGAAGCGACCTT
Ucp1	TGGCCTCTCAGTGGATGTG	CGTGGTCTCCCAGCATAGAAG
Elovl3	ACCTACATGAGAACGCGGAA	GTAGATGGCAAAGCACACGG
Hhip	GTGCCTCTGTAAAAAGGGCT	GACTCGAACTGTCCCAGAAACT
Gli2	GCCTCTGAGATGGAGACTTCTG	CAGAGGACAGGCCTTTTTCC
Boc	TCCAAGACGGACTCCTATGAGC	GCCAGAAATGGTCCAGTCGTCA
Notch1	ACAGTGCAACCCCCTGTATG	AGTTGTTCCGTAGCTGGTCG
Jag2	CCTGATTGGCGGCTATTACTG	CTGGCACACTTGTCGTACTC
Dll4	AGGGATGGGGAGGTCTGTTT	CCACCATTTCGACAGGGGTT
Mfng	TCACTGACAGCCCAGATGAACG	CCACTGACCAAGAAGGCATCGA
Ppia	GTGGTCTTTGGGAAGGTGAA	TTACAGGACATTGCGAGCAG
